# Complete chloroplast genome of *Arundina graminifolia* (*Orchidaceae*)

**DOI:** 10.1080/23802359.2019.1660281

**Published:** 2019-09-06

**Authors:** Ye Ai, Tai-Xiang Xie, Ding-Kun Liu, Xiong-De Tu, Jie Zhou, Zhong-Jian Liu

**Affiliations:** Key Laboratory of National Forestry and Grassland Administration for Orchid Conservation and Utilization, College of Landscape Architecture, Fujian Agriculture and Forestry University, Fuzhou, PR China

**Keywords:** *Arundina graminifolia*, orchid, chloroplast genome, phylogenetic analysis

## Abstract

*Arundina graminifolia* is a popular terrestrial orchid in Southeast Asia. It has high medicinal and ornamental value. In this study, the chloroplast genome of *A. graminifolia* was determined from BGISEQ-500 sequencing data. The total chloroplast genome was 159,482 bp in length, consisting of a large single-copy region (LSC 87,285 bp), a small single-copy region (SSC 18,581 bp), and two inverted repeat regions (IRA and IRB 26,813 bp). The complete chloroplast genome contains 135 genes, including 88 protein-coding genes, 38 transfer RNA (tRNA) genes, and 8 ribosomal RNA (rRNA) genes. In addition, the phylogenetic analysis indicates that *A. graminifolia* was sister to *Bletilla ochracea*, *Bletilla striata*, and *Neottia fugongensis.* The chloroplast genome will contribute to the research and conservation of *A. graminifolia*.

*Arundina graminifolia*, with a trivial name bamboo orchid, is a perennial terrestrial orchid distributed in Southeast Asia (Chen and Stephan 2009; Auberon et al. 2016). It usually grows on grassy slopes, along ravines, under shrubs or in forests at altitudes of 400–2800 m (Chen and Stephan 2009). *Arundina graminifolia* has a high ornamental value due to its beautiful flowers and long flowering time. Besides, it is one of the most common drugs used in the Dai people of China, for its extracts contains a variety of compounds such as phenols, flavonoids, and stilbenoids, which have anti-tumour, anti-virus, antioxidant, and other pharmacological activities (Liu et al. 2017). Unfortunately, with the over-excavation of *A. graminifolia*, their habitats have been broken up and the wild resources have decreased significantly over the last decades. Therefore, the *A. graminifolia* is listed as an endangered species in the China Species Red List (Wang and Xie 2004). Therefore, we report the complete chloroplast genome of *A. graminifolia*, in order to better understand the relationship between *A. graminifolia* and related genera, and contribute to the effective conservation strategy of *A. graminifolia*.

In this study, the complete genomic DNA was extracted from fresh leaves using a modified Cetyltrimethylammonium Ammonium Bromide (CTAB) method (Doyle and Doyle 1987) and sequenced by the BGISEQ-500 platform. The samples were collected from Gushan Valley, Gushan natural scenic spot, Fujian, China (26°03′N, 119°24′E) and the voucher specimen deposited at Herbarium of Fujian Agriculture and Forestry University (specimen code ZYL-GS).

The clean reads were used to assemble the complete chloroplast genome by the GetOrganelle pipe-line (Jin et al. 2018), with the chloroplast genome of *Bletilla striata* (NC_028422) as the reference sequences. The assembled chloroplast genome was annotated using the Geneious R11.15 (Kearse et al. 2012). The physical map of the chloroplast genome was generated using the online tool OGDRAW (Lohse et al. 2013). Finally, we obtained a complete chloroplast genome of *A. graminifolia* and submitted to GenBank with accession number MN_171408.

The total chloroplast genome of *A. graminifolia* was 159,482 bp in length, containing a large single-copy region (LSC 87,285 bp), a small single-copy region (SSC 18,581 bp), and two inverted repeat regions (IRA and IRB 26,813 bp). The complete chloroplast genome of *A. graminifolia* contains 135 genes, including 88 protein-coding genes, 38 transfer RNA (tRNA) genes, and 8 ribosomal RNA (rRNA) genes. The GC content of the chloroplast genome of *A. graminifolia* is 37.1%.

To investigate the phylogenetic position of *A. graminifolia*, a phylogenetic analysis was performed based on 13 complete chloroplast genome sequences of *Orchidaceae*. All sequences were aligned with the HomBlock pipeline (Bi et al. 2018) and subsequently checked manually in Bioedit version 5.0.9 (Hall 1999). Then, the phylogenetic tree constructed by RAxML (Stamatakis 2014) with 1000 ultrafast bootstrap (UFBoot) replicates (Minh et al. 2013; Chernomor et al. 2016). The results showed that *A. graminifolia* was sister to *Bletilla ochracea*, *B. striata*, and *Neottia fugongensis* with 100% bootstrap support (Figure 1).

**Figure 1.**
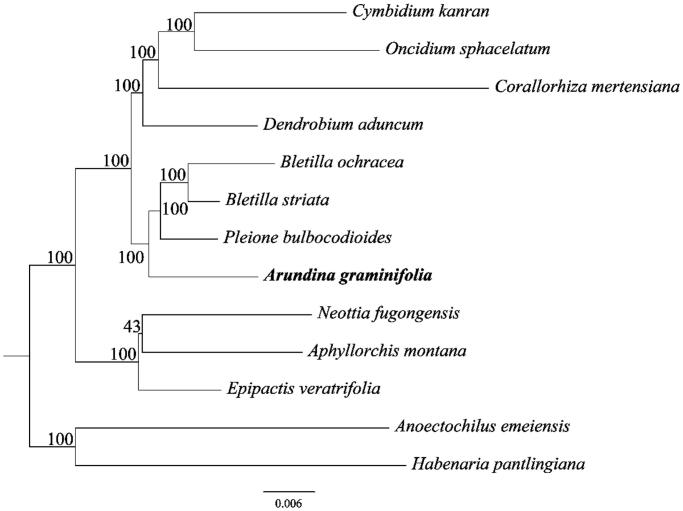
A Phylogenetic tree constructed based on 13 complete chloroplast genome sequences of *Orchidaceae*. Bootstrap support is indicated for each branch. GenBank accession numbers: *Cymbidium kanran* (NC_029711), *Oncidium sphacelatum* (NC_028148), *Corallorhiza mertensiana* (NC_025661), *Dendrobium aduncum* (NC_038077), *Bletilla ochracea* (NC_029483), *Bletilla striata* (NC_028422), *Pleione bulbocodioides* (NC_036342), *Arundina graminifolia* (MN_171408), *Aphyllorchis montana* (NC_030703), *Epipactis veratrifolia* (NC_030708), *Anoectochilus emeiensis* (NC_033895), *Habenaria pantlingiana* (NC_026775), and *Neottia fugongensis* (NC_030711).
